# Age-based prediction of uncuffed tracheal tube size in children to prevent inappropriately large tube selection: a retrospective analysis

**DOI:** 10.1186/s12871-019-0818-3

**Published:** 2019-08-07

**Authors:** Hiroshi Hanamoto, Hiroharu Maegawa, Mika Inoue, Aiko Oyamaguchi, Chiho Kudo, Hitoshi Niwa

**Affiliations:** Department of Dental Anesthesiology, Osaka University Graduate School of Dentistry, 1-8 Yamada-Oka, Suita, Osaka, 565-0871 Japan

**Keywords:** Anesthesia, Intubation, Oral surgery, Pediatric surgery, Trachea

## Abstract

**Background:**

This study aims to validate our previously reported prediction technique for uncuffed tracheal tube (TT) sizes in children younger than 2 years of age based on a calculated outer diameter (OD_Cal_, mm) in each patient according to the regression equation OD_Cal_ = 0.00223 × age (day) + 4.88 and to investigate a better method to select initial TT sizes to decrease re-intubation frequency, especially since large tubes can damage the trachea.

**Methods:**

We included patients younger than 2 years of age who underwent oral surgery under general anesthesia with tracheal intubation between July 2011 and December 2016 at the Osaka University Dental Hospital. The OD of the actual TT and the age in days were extracted from anesthesia records. Agreement rates, estimated numbers of required tubes, and size reduction frequencies were compared to obtain recommended OD (OD_Rec_) values in 2 selection groups: “average selection” in the range “nearest to the OD_Cal_ value (OD_Cal_ - 0.35 < OD_Rec_ ≤ OD_Cal_ + 0.35)” and “safe selection” in the range “nearest to the value below OD_Cal_ (OD_Cal_ - 0.7 < OD_Rec_ ≤ OD_Cal_)”.

**Results:**

The agreement rates for an OD_Rec_ in the average selection and safe selection groups were 60.8 and 55.1%, respectively (*P* = 0.001). The estimated number of required tubes per patient were 1.40 ± 0.51 and 1.47 ± 0.55 (*P* < 0.001), respectively. The estimated frequencies of size reductions were 13.3 and 4.0% (*P* < 0.001), respectively.

**Conclusions:**

Because the size reduction frequency is lower despite a slightly higher number of required TTs, selecting an OD_Rec_ based on “safe selection” parameters is desirable to avoid complications due to intubation with larger TTs.

## Background

In traditional pediatric airway management, uncuffed tracheal tubes (TTs) have been the gold standard for intubation in children under 8 years of age [1]. However, cuffed TTs are also used in pediatric practice [2]. Although cuffed TTs reduce the TT exchange rate, they do not affect the risk of complications compared with uncuffed TTs [3, 4]. Moreover, because ultrathin polyurethane Microcuff Pediatric Tracheal Tubes™ are expensive, uncuffed TTs are still regularly used in younger children in some hospitals.

Although age, height, and weight have been considered as accurate predictors of TT size, [5] the standard metric for determining it in pediatric patients younger than 2 years is controversial. Ultrasonography- [6–8] and radiography- [9] based predictions of TT sizes have been investigated in recent studies. Although these methods might well predict the actual TT size, using ultrasonography is cumbersome in some cases. Furthermore, chest radiography is not always successful because of an infant’s movements, and the risk/benefit ratio of exposing children to X-rays should be considered. Therefore, even though the current age-based prediction method might be inferior to ultrasonography or radiography correlations, it might still be the most common method in clinical practice. We previously focused on uncuffed TT size in pediatric patients younger than 2 because Cole’s formula [internal diameter (mm) = 0.25 × (age in years) + 4], which is commonly used, cannot be applied in such cases that require TT smaller than 4.0 mm. Therefore, we previously reported a regression formula for the outer diameter (OD) based on age in days [calculated OD (OD_Cal_) = 0.00223 × age (days) + 4.88, *R*^2^ = 0.511] based on data from 1035 general anesthesia cases between February 2003 and June 2011 in patients younger than 2 years [10].

Although commercially available TTs are made with an inner diameter of 0.5 mm, the OD differs depending on the type and manufacturer. However, a TT completely consistent with the OD_Cal_ rarely exists in a clinical setting, especially since commercially available TTs have inner diameter increments of 0.5 mm, resulting in OD differences of approximately 0.7 mm [7]. Even if anesthesiologists use a regression equation and select the TT nearest to the OD_Cal_, there is a risk of intubating with a TT that is too large and might damage the trachea or the vocal cord. Therefore, we hypothesized that slightly smaller TT sizes might be desirable to prevent an inappropriately large tube selection. The purpose of this study was to validate uncuffed TT size predictions based on the patient’s age in days and to investigate a better method to select TTs to decrease the frequency of size reduction and repeated intubation in children under 2 years of age.

## Methods

This study was approved by the Ethics Committee at Osaka University Graduate School of Dentistry (approval number: H30-E1). The requirement for written informed consent was waived. Patients aged less than 2 years who underwent oral surgery under general anesthesia and required tracheal intubation between July 2011 and December 2016 at our hospital were included in this study. Exclusion criteria were the use of cuffed TTs and tracheostomies. The anesthesia records of patients were retrospectively investigated. The OD of the used TT (mm) and the age (day) were recorded. We used the OD as the TT size because ODs are different depending on the type and manufacturer of the TT.

### Anesthesia and selection of tracheal tube size

Anesthesia was induced with sevoflurane, thiamylal, or propofol. After anesthesia induction, neuromuscular blocking agents were administered before intubation. Although we obtained a regression equation based on our previous data, the TT size on the first attempt was selected at the discretion of the attending anesthesiologists. An adequate TT fit was judged based on air leakage. A leak test was performed after each tracheal intubation by increasing respiratory pressure gradually up to approximately 35 cm H_2_O, and 1 or 2 senior anesthetists listened for an audible leak sound near the patient’s mouth. At our institution, an uncuffed tube was considered an appropriate size for oral surgery when no air leaks were observed at less than 15 cm H_2_O; this threshold prevents intraoral blood from flowing into the trachea. If an air leak was observed with an inflation pressure of less than 15 cm H_2_O, the next largest TT size available was chosen. When no air leak was observed at 35 cm H_2_O and there was resistance during TT insertion, the intubated TT was exchanged for one with a smaller OD. In cases of no resistance, the original TT choice was used.

### Validation analysis

We plotted the scatter diagram between the OD and age and drew the regression line obtained by our previous study. According to this regression equation, an OD was calculated for each patient (OD_Cal_, OD_Cal_ = 0.00223 × day + 4.88). However, because a TT with the estimated OD_Cal_ does not always exist, a recommended OD (OD_Rec_, OD of the first selected TT) was considered. To determine OD_Rec_ values in the range nearest to or below OD_Cal_ values, we also added lines parallel to the regression line at intervals of 0.7 mm because most commercially available uncuffed TTs have OD differences of approximately 0.7 mm [7]. These parallel lines were created using 1 of 2 methods: in the first method, we created lines ±0.7 and ± 1.4 mm from the regression line; in the second method, the parallel lines were created ±0.35 and ± 1.05 mm away from the regression line. The OD_Rec_ value was then compared with the OD of the actual TT used in each patient.

### Outcomes

The outcomes were the agreement rate between OD and OD_Rec_ values in the total number of cases, the estimated number of required tubes per patient, and the estimated frequency of size reduction. We defined “average selection” as that in the range nearest to the OD_Cal_ value (OD_Cal_ - 0.35 < OD_Rec_ ≤ OD_Cal_ + 0.35) and compared it to the “safe selection” calculation, which was in the range nearest to the value below OD_Cal_ (OD_Cal_ - 0.7 < OD_Rec_ ≤ OD_Cal_). The standard value provided by the manufacturers was used as the OD of each type and size of TT.

### Statistical analysis

The sample size was determined by the study period. The start of the study period was determined as after our previous study period (which was between February 2003 and June 2011) [10]. The end of the study period was December 2016, because cuffed tubes have been used since 2017 at our institution. All statistical analyses were performed with EZR (Saitama Medical Center, Jichi Medical University, Saitama, Japan), which is a graphical user interface for R software (The R Foundation for Statistical Computing, Vienna, Austria). More precisely, this program is a modified version of the R commander that was designed with statistical functions frequently used in biostatistics [11]. Continuous variables are presented as the mean ± standard deviation (SD) with comparisons performed using a paired t-test. Dichotomous or categorical variables are presented as numbers (percentages) and were analyzed using the McNemar’s test. A *P*-value < 0.05 indicated statistical significance.

## Results

Uncuffed TTs from different manufacturers were used in this study. The ODs of these TTs with their corresponding inner diameters are shown in Table 1. Although the “Spiral” tube type was not used during the study period, we have stated its parameters in the table because it was included in our previous study [10]. Severe, adverse respiratory events including severe postoperative croup or subglottic edema were not observed.

**Table 1:** Outer diameters of uncuffed tracheal tubes from different manufacturers according to their inner diameters

Inner diameter (mm)	Outer diameter (mm)			
	SILICONISED	IVORY	RAE	SPIRAL
3.0	4.2	4.4	4.2	4.7
3.5	4.8	5.1	4.8	5.3
4.0	5.5	5.9	5.6	6.0
4.5	6.2	6.6	6.2	6.7
5.0	6.9	7.3	6.9	7.3
5.5	7.6	8.0	7.4	8.0

SILICONISED: Portex siliconized PVC, uncuffed tracheal tube

IVORY: Portex tracheal tube IVORY, uncuffed tracheal tube

RAE: Mallinckrodt oral RAE tracheal tube, uncuffed, Murphy eye

SPIRAL: PHYCON wire reinforced uncuffed tube

**Fig. 1:**
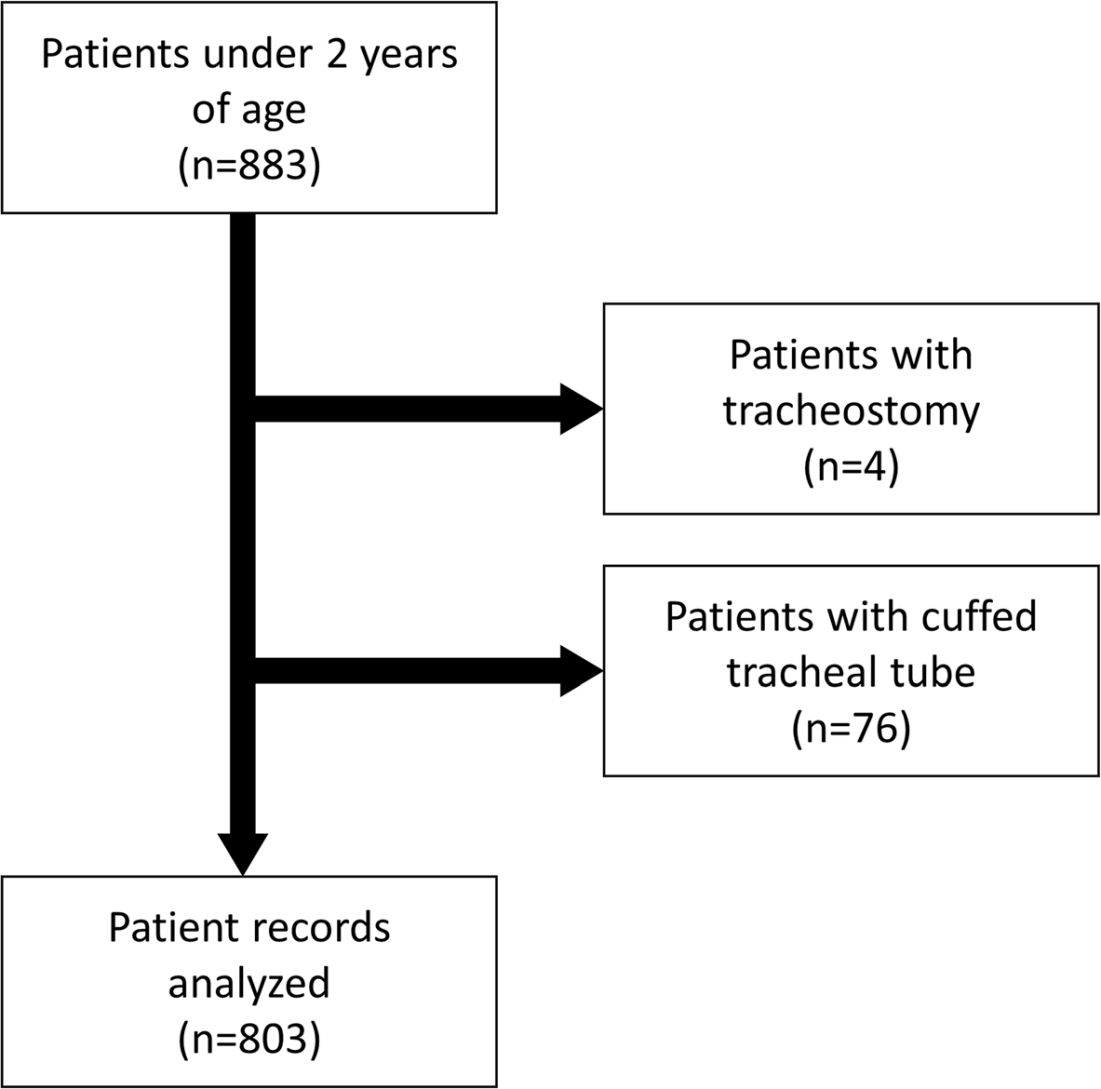
Flow chart of patient selection

**Fig. 2:**
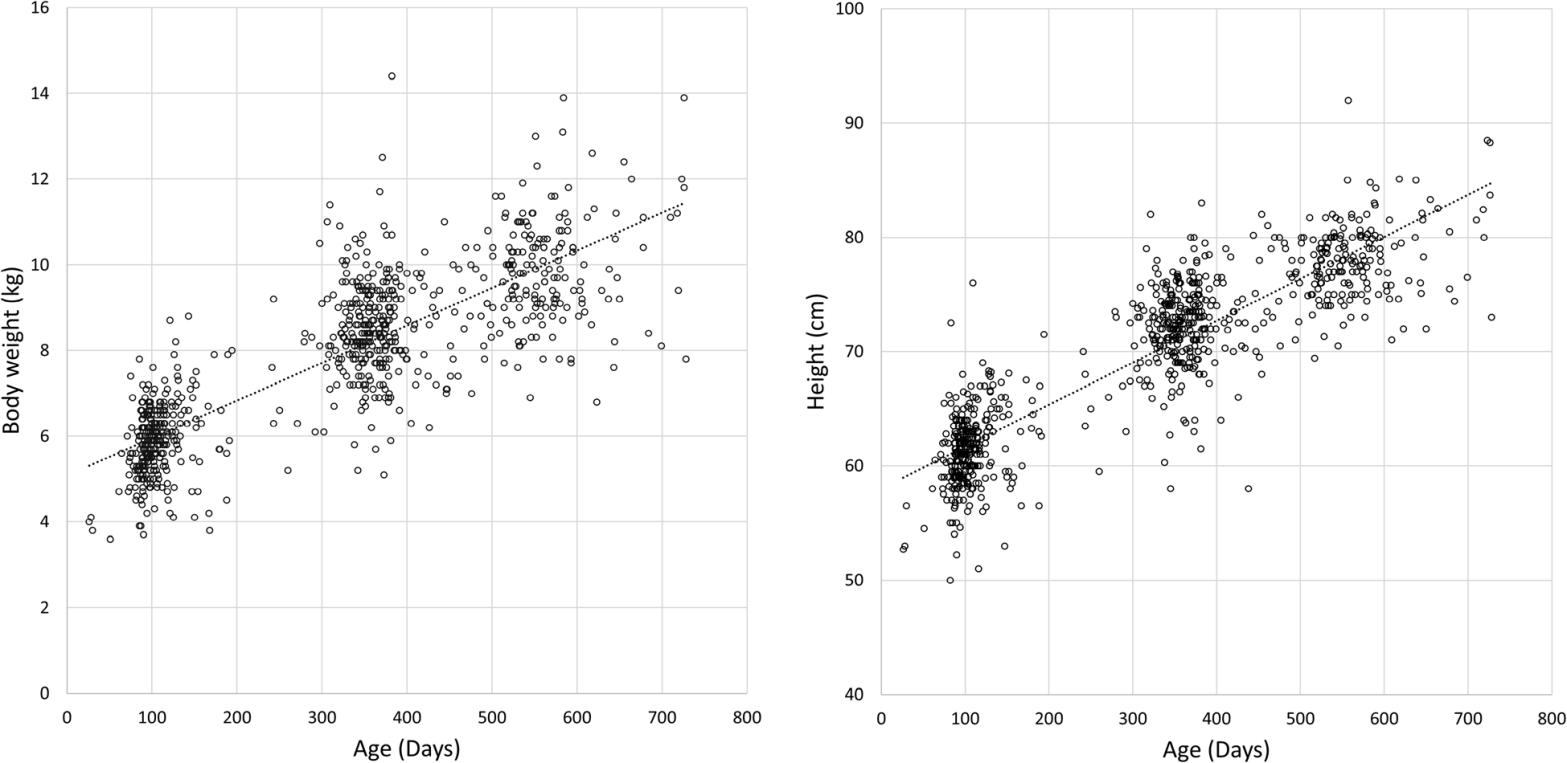
Scatterplot of age in relation to height and body weight. Line in the scatter plot are as follows: dotted line, regression line

We reviewed 883 anesthetic records of patients under 2 years of age during the study period. Four cases of tracheostomy and 76 cases with cuffed TT use were excluded. A total of 803 anesthetic records without any missing data were finally analyzed (Fig. 1). Demographic data are presented in Table 2. Patient age in relation to height or body weight are presented in Fig. 2.

**Table 2:** Demographic and procedural data

Variables	Values
Age, days	308 ± 182
Body weight, kg	7.8 ± 1.9
Height, cm	69.3 ± 7.6
Sex, M / F	459 / 344
Main operation method	
Cheiloplasty	308 (38.4%)
Palatoplasty	454 (56.5%)
Tongue operation	34 (4.2%)
Lip repair	3 (0.4%)
Other oral surgery	4 (0.5%)
Operation time, min	68 ± 24
Anesthesia time, min	138 ± 27

Data are presented as number of patients (percentage) or mean ± standard deviation. *M* Male, *F* Female

**Fig. 3:**
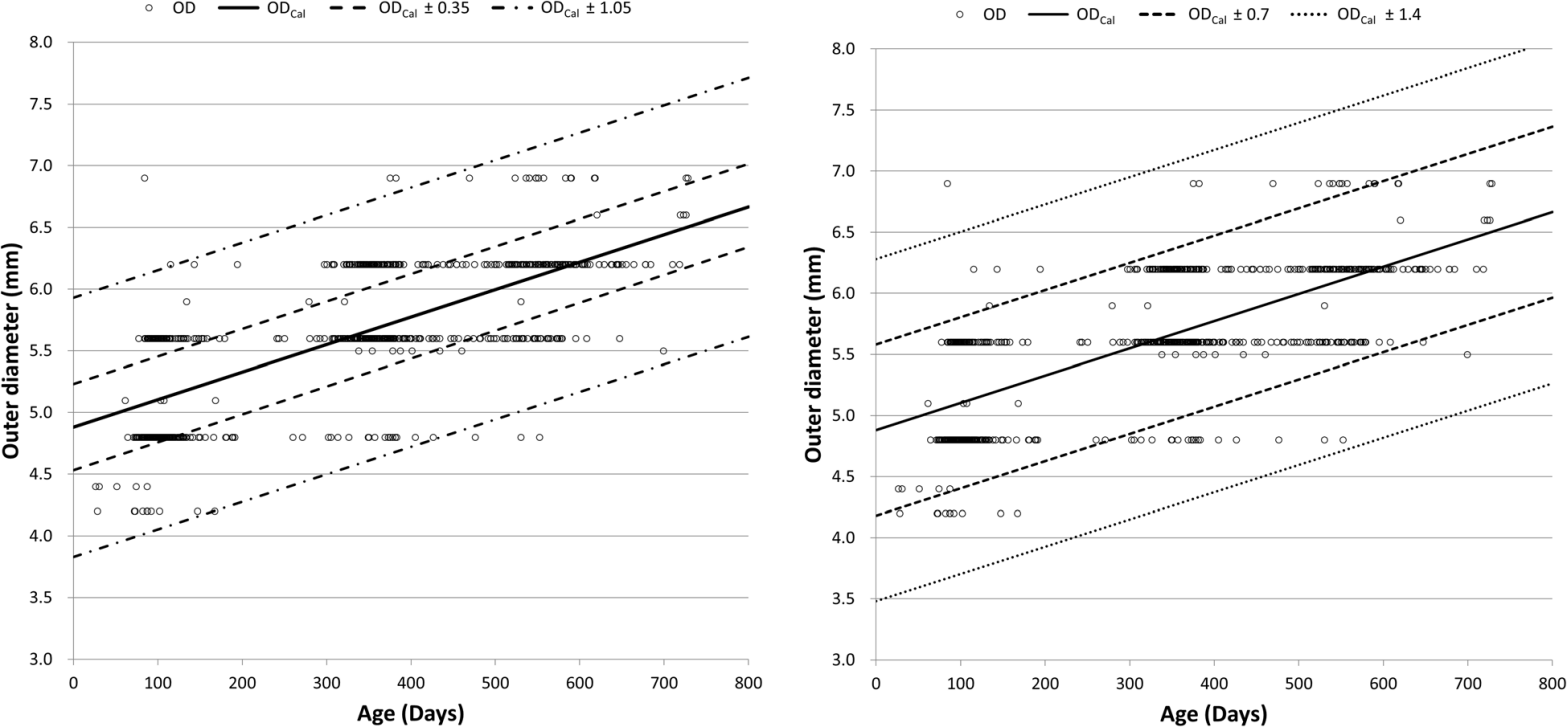
Scatterplot of age in relation to the outer diameter of the tracheal tube used. Lines in the scatter plot are as follows: solid line, regression line; long-dashed lines, regression line ±0.35 mm; and dashed-dotted lines, regression line ±1.05 mm, regression line ±0.7 mm; and dotted lines, regression line ±1.4 mm. OD, outer diameter of the tube used, mm; OD_Cal_, calculated outer diameter, mm

Parallel lines to the regression line of OD_Cal_ in the scatter plot of OD are shown in Fig. 3. The estimated numbers of required TTs per patient in the average selection and safe selection groups are shown in Tables 3 and 4, respectively. The agreement rates were 60.8 and 55.1% (*P* = 0.001), the estimated numbers of required TTs per patient were 1.40 ± 0.51 and 1.47 ± 0.55 (*P* < 0.001), and the estimated probabilities of a required size reduction were 13.3 and 4.0% (*P* < 0.001), respectively.

**Table 3:** Number of cases and estimated number of required tubes per patient with the predicted outer diameter determined by “nearest value to the calculated outer diameter”

OD of tracheal tube	Number of cases (%)	Estimated number of required tubes
OD ≤ OD_Cal_ - 1.05	4 (0.5)	3
OD_Cal_ - 1.05 < OD ≤ OD_Cal_ - 0.35	103 (12.8)	2
OD_Cal_ - 0.35 < OD ≤ OD_Cal_ + 0.35	488 (60.8)	1
OD_Cal_ + 0.35 < OD ≤ OD_Cal_ + 1.05	204 (25.4)	2
OD_Cal_ + 1.05 < OD	4 (0.5)	3

*OD* Outer diameter of the used tube, mm

*OD*_*Cal*_ Calculated outer diameter, mm

Estimated number of required tubes, estimated number of required tubes per patient when the recommended outer diameter of the first selected tracheal tube (OD_Rec_) was assumed to be in the range “OD_Cal_ - 0.35 < OD_Rec_ ≤ OD_Cal_ + 0.35”

**Table 4:** Number of cases and estimated number of required tubes per patient with the predicted outer diameter determined by “nearest value below the calculated outer diameter”

OD of the tracheal tube	Number of cases (%)	Estimated number of required tubes
OD ≤ OD_Cal_ - 1.4	0 (0)	3
OD_Cal_ - 1.4 < OD ≤ OD_Cal_ - 0.7	31 (4.0)	2
OD_Cal_ - 0.7 < OD ≤ OD_Cal_	442 (55.1)	1
OD_Cal_ < OD ≤ OD_Cal_ + 0.7	312 (38.8)	2
OD_Cal_ + 0.7 < OD ≤ OD_Cal_ + 1.4	17 (2.0)	3
OD_Cal_ + 1.4 < OD	1 (0.1)	4

*OD* Outer diameter of the used tube, mm

*OD*_*Cal*_ Calculated outer diameter, mm

Estimated number of required tubes, estimated number of required tubes per patient when the recommended outer diameter of the first selected tracheal tube (OD_Rec_) was assumed to be in the range “OD_Cal_ - 0.7 < OD_Rec_ ≤ OD_Cal_”

## Discussion

Our findings support the selection of an OD_Rec_ in the “safe selection” range (OD_Cal_ - 0.7, OD_Cal_). Our size reduction probability was 4% with an acceptable estimated number of required TTs per patient, slightly higher than that needed for the “average selection” range (OD_Cal_ - 0.35, OD_Cal_ + 0.35). To our knowledge, this is the first report on the number of required TTs and the probability of a required size reduction after TT selection. Although the estimated number of required TTs was lower in the “average selection” group, we believe that the “safe selection” method is adequate based on intubation times and the probability of a required size reduction.

Previously reported agreement rates were 48% [7], 60% [8], and 98% [6] predicted by ultrasonography, 57% by radiography [9], and 52.6% using middle finger length [12]. Agreement rates of age-based formulae vary widely with values of 53.5% [13], 60% [6], 24–40% [7], and 32–43% [9]. In the present study, the agreement rate was 60.8% when the first selection used the “average selection” approach and 55.1% when predicted by the “safe selection” method; these values are not low compared to other studies. Eck et al., who had conducted one of the few previous studies on the size of uncuffed TTs in patients younger than 2 years, calculated a regression equation to predict TT sizes for infants younger than 1 year [14] with an R^2^ value of 0.387, indicating poor correlation.

Uncuffed TT selection should incorporate the patient’s benefit, economic factors, and the anesthetist. Considerations for the patient’s benefit should include a decreased frequency of laryngoscopy and intubation, as well as preventing intubation with an oversized TT. From an economical perspective, it is better to reduce the number of TTs used. With an OD_Rec_ determined by “safe selection” parameters, the average number of required TTs was 1.47. Uncuffed TTs may have a beneficial economic effect; in fact, a Microcuff™ tube in our country is approximately 2.8-fold more expensive than an uncuffed tube.

Some anesthetists perform re-intubation with the next smallest TT if the primarily selected TT does not exhibit an air leak. If the consequent leak is too large and ventilation becomes difficult, re-intubation is performed again using the first TT. In this situation, the anesthetist has confirmed an excessive air leak when using the smaller TT, but the frequency of laryngoscopy and intubation has been increased. Although using an airway catheter is one of the methods for tube exchange and prevention of laryngoscopy, it is difficult to judge the insertion resistance. Another anesthetist may use the first TT to avoid re-intubation if neither an air leak nor insertion resistance is present. In this situation, anesthesia experience is required, and an anesthetist may be apprehensive regarding TT use; therefore, a smaller TT may be optimal. In consideration of these issues, smaller sized TTs based on safe selection parameters should be primarily selected.

Generally, if there is no air leak at an inflation pressure of 25–40 cm H_2_O, a TT should be substituted with a smaller sized one [1, 15]. However, we used the criterion of the lack of an air leak at less than 15 cm H_2_O as a guide for appropriate TT selection as this threshold prevents intraoral blood from flowing into the trachea during surgery. Therefore, there might be several cases in our study in which an air leak would not have been observed at an inflation pressure of 25–40 cm H_2_O. In adults, tracheal mucosal capillary blood flow is compromised above 30 cm H_2_O and totally obstructed above 50 cm H_2_O [16]. Adverse events increase in children with no air leaks at 25 cm H_2_O [17]. In the present study, severe respiratory adverse events were not observed.

Previous studies adopted several ranges as criteria for appropriate leak pressure including 10–30 cm H_2_O [9, 14], 10–20 cm H_2_O [6], 15–25 cm H_2_O [7], 15–30 cm H_2_O [8], 10–35 cm H_2_O [18], and 5–40 cm H_2_O [19]. This indicates that the optimal leak pressure is still controversial. Also, there were some cases in which the optimal TT size that met the leak pressure criteria was not found. Furthermore, results may differ according to the chosen air leak pressure range. In the present study, all patients underwent oral surgery accompanied with intraoral bleeding. Therefore, it is important to use a TT with an optimal fit to prevent air leaks into the trachea. This air leak criterion may have led to the selection of slightly larger TTs in our study when compared with other studies. In general intubation situations, a slightly smaller size might be better based on the air leak criteria, although our data are especially applicable to a variety of patients undergoing oral surgery. Particularly in difficult airway situations [20], reduction of TT exchange and selection of excessively large TT are required.

### Limitations

This study has several limitations. First, the head position might affect the leak pressure [21]. Our results included surgeries performed with an extended head, such as palatoplasty in cleft palate patients, but the head position during surgery was not the same in all cases. Second, most ODs of commercially available TTs are at increments between 0.6 and 0.8 mm. We calculated the estimated number of required TTs using the value of 0.7 mm as the difference in size between different ODs. Therefore, our results may be subject to error or potential bias. However, we consider such error to be acceptable, because we used a mean value for generalization. The third limitation is the retrospective design of our study; consequently, actual intubation times were not recorded in all cases. Hence, further prospective studies are needed. Although there are some limitations, these study findings can improve TT selection under various conditions. Moreover, if future material improvements make it possible to further decrease the thickness of the TT wall, our results may be directly applicable to the prediction of uncuffed TT sizes.

## Conclusions

Uncuffed TT size predictions based on the range “OD_Cal_ – 0.7 < OD_Rec_ ≤ OD_Cal_” in patients under 2 years of age is easy and comprehensively adequate because the probability of size reduction is less than 4% and the number of required TTs and laryngoscopies are low. Even if their chosen criteria for TT air leaks are different, anesthesiologists should select a TT size for the first attempt based on this “safe selection” approach using the “nearest value below OD_Cal_” rule according to the regression equation obtained at each institution, as this rule is superior to the “nearest to OD_Cal_” rule.

## Data Availability

The datasets of this study can be obtained from the corresponding author upon reasonable request.

## Abbreviations

OD: Outer diameter of the tube used
OD_Cal_: Calculated outer diameter
OD_Rec_: Recommended outer diameter
TT: Tracheal tube
